# Neonatal sepsis and transient immunodeficiency: Potential for novel immunoglobulin therapies?

**DOI:** 10.3389/fimmu.2022.1016877

**Published:** 2022-10-18

**Authors:** Coco R. Beudeker, Daniel C. Vijlbrief, Joris M. van Montfrans, Suzan H.M. Rooijakkers, Michiel van der Flier

**Affiliations:** ^1^ Department of Pediatric Infectious Diseases and Immunology, University Medical Center Utrecht, Utrecht, Netherlands; ^2^ Department of Neonatology, University Medical Center Utrecht, Utrecht, Netherlands; ^3^ Department of Medical Microbiology, University Medical Center Utrecht, Utrecht, Netherlands

**Keywords:** immunodeficiency, neonatal sepsis, monoclonal antibodies, complement system, neutrophil function, immunoglobulin therapy

## Abstract

Neonates, especially preterm neonates, have the highest risk of sepsis of all age groups. Transient immaturity of the neonatal immune system is an important risk factor. Neonates suffer from hypogammaglobulinemia as nor IgA nor IgM is transferred over the placenta and IgG is only transferred over the placenta late in gestation. In addition, neutrophil numbers and complement function are also decreased. This mini-review focuses on strategies to improve neonatal host-defense. Both clinical and preclinical studies have attempted to boost neonatal immunity to lower the incidence of sepsis and improve outcome. Recent advances in the development of (monoclonal) antibodies show promising results in preclinical studies but have yet to be tested in clinical trials. Strategies to increase complement activity seem efficient *in vitro* but potential disadvantages such as hyperinflammation have held back further clinical development. Increase of neutrophil numbers has been tested extensively in clinical trials but failed to show improvement in mortality. Future research should focus on clinical applicability of promising new prevention strategies for neonatal sepsis.

## Introduction

Neonatal sepsis is a global health burden, with 1,4 million cases and 200,000 deaths worldwide each year (1). Preterm infants (born at <37 weeks of gestational age) are particularly at risk of sepsis (2). Despite continuous efforts to improve survival in this vulnerable patient population, susceptibility to and severity of bacterial infections remain disproportionate compared to other age groups. This susceptibility is multifactorial: transient immunodeficiency of immaturity, indwelling central lines and respiratory support are important risk factors (3). This review focuses on the physiological transient immaturity of the neonatal immune system.

Both innate and adaptive immunity are immature in neonates. We focus on three major components of the immune system that are impaired in neonates. Firstly, neonates suffer from transient antibody deficiency, reflecting immaturity of the adaptive immune system (4). Secondly, complement levels are low in preterm neonates, when compared to term neonates and adults (5). Thirdly, preterm neonates have lower absolute neutrophil counts at birth, and some neutrophil functions may be decreased (6). Both complement and neutrophils reflect immaturity of the innate immune system. These three factors contribute to the high risk of sepsis in neonates, which is most pronounced in preterm neonates.

The importance of host defense in neonatal infection is illustrated by neonatal sepsis as presenting symptom of inborn errors of immunity, which may manifest at a young age due to limited redundancy in the immature immune system (7).

This review provides an overview of several available and novel strategies that aim to prevent or treat neonatal sepsis by harnessing the host response. We discuss laboratory studies and clinical trials results and address the latest advances in host targeted therapies, primarily focusing on novel immunoglobulin therapies (see **Supplementary Table 1** and **Supplementary Table 2** for an overview of the clinical and preclinical studies reviewed).

## Hypogammaglobulinemia

Immunoglobulins are an essential component of the adaptive immune system. They recognize and bind antigens and are able to activate the complement system and immune cells of the innate immune system, such as neutrophils. Immunoglobulins can be classified into five different subtypes (IgA, IgD, IgE, IgG and IgM), which differ in antigen recognition and effector function (8). Lower levels of immunoglobulins (hypogammaglobulinemia), can lead to both recurrent infections and severe infections, resulting in an increased mortality (9).

Fetal and neonatal antibody production is low. Therefore, neonates depend on transplacental maternal antibody transfer to provide protective plasma immunoglobulin levels against pathogens. Transplacental antibody transfer is restricted to IgG and occurs through active transport by the neonatal Fc receptor (FcRn) (10). IgA and IgM do not bind to the FcRn. As a result, all newborns are deficient in IgA and IgM (11).

**Figure 1:**
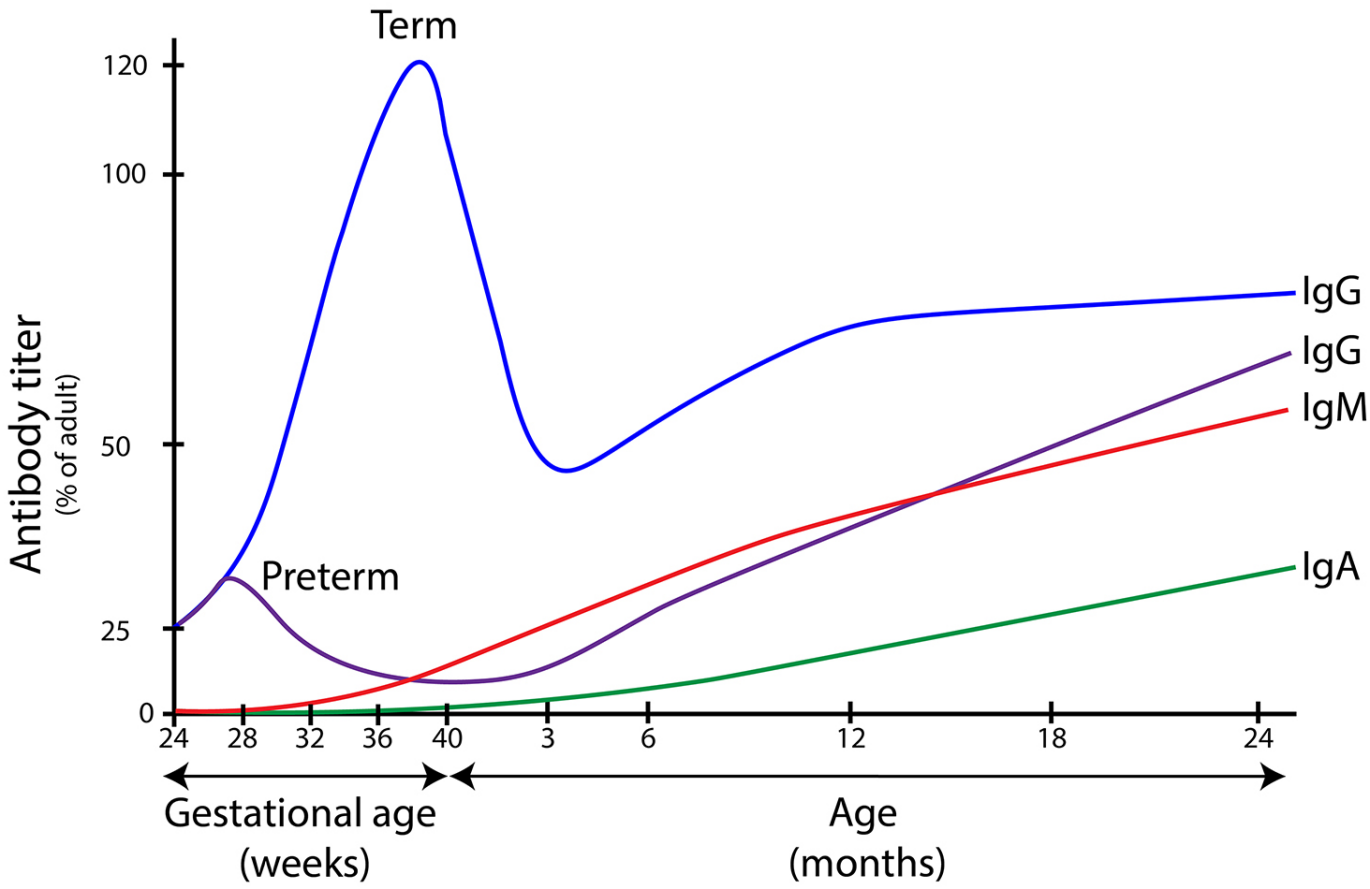
Immunoglobulin serum concentrations in different age groups. Levels of immunoglobulins A, G and M in different stages of life. IgG is actively transported over the placenta during pregnancy, with a peak between 36 and 40 weeks of gestational age. Children born before this age have lower levels of IgG at birth and are at increased risk for bacterial infections. In new born infants, IgG wanes over time which leads to a window of increased susceptibility to infection (between the age of 3 months and 2 years in term infants). IgA and IgM are not transferred over the placenta, which means that children are dependent on endogenous antibody production to reach protective serum IgA and IgM concentrations. Immunoglobulin levels are depicted as percentage of average adult serum levels (median adult IgG 10.9 g/L; median adult IgA 1.3 g/L median adult IgM 2.3 g/L. The figure is a composite of information from many sources (12–17).

Transplacental transfer of IgG starts around 13 weeks of gestational age and fetal IgG levels rise to about 10% of maternal IgG levels at 22 weeks, 50% at 32 weeks, and spike in the final four weeks of pregnancy (**Figure 1**) (12–17). Since most IgG are transferred after 36 weeks of gestational age, preterm neonates born before that time have deficient plasma IgG levels and are at especially increased risk of sepsis (18). Additionally, endogenous antibody production is low up to 12 months of age, thus term neonates also face an increased susceptibility to infectious diseases as antibody production initially does not compensate antibody degradation rates (19).

### Intravenous immunoglobulin G

An obvious strategy to protect newborns vulnerable to infection due to hypogammaglobulinaemia is antibody supplementation therapy in the form of intravenous immunoglobulins (IVIG) (20). IVIG consists of pooled immunoglobulins (mainly IgG) of at least 1000 healthy donors and is administered intravenously or subcutaneously (21). Antibody replacement therapy effectively prevents infections in patients with inborn errors of immunity characterized by hypogammaglobulinaemia (22). The effectiveness of IVIG as prophylaxis for sepsis in neonates has been studied extensively in randomized controlled trials (RCTs) in preterm and low birth weight neonates (birth weight < 2500 gram). IVIG administration is safe and resulted in a modest 3% reduction in sepsis and a 4% reduction in one or more episodes of any serious infection but was not associated with reductions in other clinically relevant outcomes, including mortality (23). Potentially, IVIG could be used therapeutically to treat sepsis. However, thus far, the use of IVIG to treat sepsis in addition to antibiotics showed no benefit (24).

The disappointing results with neonatal IVIG therapy may in part be attributed to a high variability in concentration of antibodies against relevant pathogens between batches of IVIG (25). Enrichment of IVIG with IG’s against common neonatal pathogens may increase efficacy.

### Intravenous immunoglobulin G enriched with IgM and IgA

IgM is more efficient than IgG in activating the complement system. As IgM does not pass the placenta, newborns are deficient in IgM, and as such, administering IgM to newborns seems like a rational option to improve immunity (26). However, studies conducted with the currently available IgM-enriched IVIG preparation (Pentaglobin), did not show improved outcomes in terms of mortality or morbidity (24). These disappointing results could be due to the treatment of Pentaglobin with ß-propiolactone to decrease immunoglobulin aggregation. However, this also leads to complement fixation and reduced Fc-binding capacity of immunoglobulins (27). The recently developed IgM-enriched preparation Trimodulin is manufactured differently. It is not treated with ß-propiolactone and contains twice the amount of IgA (21%) and IgM (23%) in comparison to Pentaglobin, and is therefore potentially more effective (28, 29). Recently, the results of a phase II trial, which included 160 adult patients with severe community-acquired pneumonia treated with Trimodulin, were published. Although no difference was observed for ventilator-free days between the placebo and Trimodulin groups, *post-hoc* analyses supported improved outcome in patients with reduced IgM (29).

No neonatal studies with this new IgM-enriched immunoglobulin preparation have been performed to date.

### Maternal vaccination

In general, transplacental antibody transfer does not protect the child against pathogens specific for the neonatal period, as adults, including pregnant women, have low circulating antibody levels against these pathogens. Maternal vaccination strategies aim to specifically induce maternal antibodies to pathogens threatening the days and weeks after birth (30). Currently, vaccinations against several other diseases are available or under development for pregnant women, including diphtheria, tetanus, respiratory syncytial virus (RSV) and Group B streptococcus (GBS), the latter being an important cause of neonatal sepsis (31–35).

Although maternal vaccination is a promising strategy to prevent neonatal infections, it does not protect preterm neonates at the highest risk of infection since they are born before the late transplacental transfer of antibodies.

### Monoclonal pathogen-specific immunoglobulins

Because of their ability to efficiently neutralize pathogens, monoclonal antibodies (mAbs) are currently extensively researched to prevent or treat infectious diseases. Currently, several mAbs are available, the oldest US Food and Drug administration (FDA) approved one is palivizumab, a prophylactic antibody therapy against severe respiratory syncytial virus infection in preterm neonates. Against coronavirus disease 2019 (COVID-19) several mAbs have been FDA approved, for instance casirivimab and imdevimab, an antibody combination for therapeutic treatment of mild to moderate infection in children and adults with high risk of progression to severe COVID19 disease (36–38). Bezlotoxumab is a mAb targeted against bacteria and is efficient in preventing recurrent *Clostridium difficile* infection (39). These examples demonstrate the potency of mAb therapy to prevent disease or severe disease progression.

MAbs could be a promising new treatment option for neonatal sepsis. Unfortunately, the first randomized controlled trial to test a Staphylococcus-specific mAb pagibaximab (directed against anti-lipo-teichoic acid) failed to demonstrate significant reduction in staphylococcal late onset neonatal sepsis in preterm infants ≤32 weeks (40). The reasons for this failure are incompletely understood. However, mAbs are targeted at one pathogen, while many different pathogens can cause neonatal sepsis. Thus, the future of mAb based therapies to prevent neonatal sepsis likely lies in the development of a cocktail of different mAbs targeted against different pathogens. Currently, several preclinical studies examined the potential of other mAbs against common neonatal pathogens, such as *S. epidermidis, E. coli, Klebsiella spp* and *GBS* (41–45) (**Supplementary Table 2**).

### Synthetic polyvalent immunoglobulins

Passive immunization, using immunoglobulins derived from plasma of donors recently vaccinated or infected with a certain pathogen which results in a high immunoglobulin titer to that specific pathogen [polyvalent hyperimmune IgG (HIG)], is an effective strategy to protect against specific life-threatening diseases (46). An example of the successful application of HIG is the post-exposure prophylaxis with varicella-zoster immune globulin (Varizig) to prevent varicella in exposed preterm neonates (47). However, two anti-staphylococcal HIG INH-A2 (anti-staphylococcal surface components hyperimmune IgG) and Altastaph (*S. aureus* serotype 5 and 8 vaccine induced hyperimmune IgG) were not effective in randomized controlled trials to prevent staphylococcal late onset neonatal sepsis in preterm infants ≤32 weeks (48). In addition, as production of HIG is costly and slow, the demand for these antibodies will always exceed supply.

In recent years, novel techniques have emerged that allow production of synthetic polyvalent immunoglobulins (49). These are molecular genomic strategies to capture diverse mammalian antibody repertoires to create recombinant multivalent hyperimmune globulins. The antigen-specific regions can be derived from previously developed mAbs or be copied from the B cells of individuals recently vaccinated against or infected with the pathogen of interest. This mimics natural HIG preparations from donors. The benefit of polyvalent immunoglobulins is the possibility to target several different epitopes on a pathogen and target multiple different types of pathogens. This new technique could potentially increase the potency of anti-infective immunoglobulin therapy.

### Modified monoclonal antibodies

Immunoglobulins can be modified in several ways to increase their efficiency. One way to enhance immunoglobulin effector function is modification of glycosylation. Glycosylation of antibodies does not alter antigen binding but does affect downstream effector functions, such as binding of antibodies to the Fc receptor (50). For example, Rituximab, a recombinant monoclonal antibody (rMAb) directed against the B cell receptor CD19, shows more effective antibody-mediated killing in an a-glycosylated state than its glycosylated form (50, 51). Altering the glycosylation of antibodies may potentially improve antibody-mediated killing, therefore providing a better protection against pathogens.

A second way to maximize the beneficial effect of immunoglobulins is to increase the half-life. Antibody levels wane over time. Palivizumab, for example, has a half-life of approximately 20 days and needs to be administered monthly to maintain prophylactic levels (52). By modifying the Fc region of this antibody, an extended half-life antibody (nirsevimab) has been developed with a half-life of up to 100 days, allowing for less frequent dosing of prophylactic antibodies, as a bridge to adequate endogenous antibody production (53).

A third way to modify synthetic antibodies is to alter the Fc tail to facilitate polymerization of antibodies upon binding to the antigen. Hexamerization of IgG at the bacterial surface is needed to efficiently activate complement. Complement factor C1Q binds IgG hexamers and initiates the classical complement activation route. These results in opsonization of the bacterial surface with complement factor C3b and formation of the membrane attack complex (MAC) on the surface of Gram-negative bacteria. Antibodies with hexamerization-enhancing mutations (hexabodies) activate complement more efficiently compared to wild-type IgG. These hexabodies showed more efficient C3b activation and phagocytosis of *Staphylococcus aureus in vitro*, *Streptococcus pneumoniae in vitro* and more efficient MAC activation and bacterial lysis of *Neisseria gonorrhoeae in vitro* and *in vivo (* 54–56). Recently, it was shown that hexabodies can also enhance C3b activation and phagocytosis of *Staphylococcus epidermidis* in the context of neonatal plasma *in vitro (* 42).

There are different ways to alter immunoglobulins to make them more efficient in pathogen neutralization, and the three methods mentioned above can be considered when designing synthetic immunoglobulins.

## Complement deficiency

The complement system is an important element of human immune defense against bacterial infections. The killing of Gram-negative bacteria can be accomplished by the complement system alone, through lysis of the bacterial cell *via* the formation of the membrane attack complex. Gram-positive bacteria on the other hand, have thicker cell walls and no outer cell membrane and can only be killed by phagocytic cells after being coated with antibodies and complement, a process called opsonophagocytosis (57). Complement deficiencies lead to an increased risk of infections caused by both Gram-negative and Gram-positive bacteria (58). Newborns have decreased complement function compared to adults, and preterm neonates are most deficient in complement activity (5). This relative complement deficiency contributes to an increased susceptibility to infectious diseases. On the other side, over-activation of complement can lead to tissue damage in patients with severe infections, such as sepsis or meningitis. Therefore, modulation of complement (both inhibition and specific activation) has been a point of interest in immunology research.

### Enhancing complement activation in neonates

Properdin is a component of the complement system that enhances alternative pathway complement activity. The alternative complement pathway functions by amplifying complement activation resulting from the classical and lectin activation routes. Decreased levels of properdin, for example in patients with genetic defects or newborns, are associated with susceptibility to severe bacterial infections (5, 59). A possible therapy would be administering (low dose) properdin to these patients. In theory, this approach could also strengthen complement activation in response to infection in newborns. One preclinical study showed that a low dose of recombinant properdin significantly boosted resistance against *S pneumonia* and *N meningitidis* infection *in vitro* and animal infection model data (60). Application in patients of this approach carries risks, as massive complement activation in the context of sepsis may induce hyperinflammation and tissue damage (61). In the animal disease model, this hyperinflammation was not described. However, translation from animal models to clinical application is difficult, as murine models poorly reflect human inflammatory conditions (62).

Controlled modulation of complement remains a challenge, as complement inhibition could lead to infections, and complement activation can lead to hyperinflammation.

### Complement inhibition

In severe infectious or inflammatory conditions, complement plays an important role in hyperinflammation. Specific inhibition of the complement system has been used to decrease the effects of hyperinflammation in different diseases. A complement inhibitor (eculizumab) was developed to treat certain auto-inflammatory conditions, such as paroxysmal nocturnal hematuria (PNH) or atypical hemolytic uremic syndrome (aHUS). This mAb prevents cleavage of C5, which leads to decreased C3 fragmentation on red blood cells in PNH and contributes to reduced hemolysis in PNH and aHUS patients (63, 64). Recently a similar approach has been tested in patients with infection-associated hyperinflammation. For example, patients with severe COVID-19 caused by SARS-CoV-2 have severe tissue damage attributed to increased complement activity (65). One small case series described the successful use of eculizumab in pediatric acute SARS-CoV2 infection, multisystem inflammatory syndrome and thrombotic microangiopathy (66). The same principle of complement inhibition has been tested in a small phase-II clinical trial of adult patients with severe COVID-19 caused by SARS-CoV-2. To prevent hyperinflammation in these patients, a mAb that blocks C5a (vilobelimab) was administered, and preliminary results show a decrease in pulmonary embolisms (67). Vilobelimab has not yet been tested in pediatric patients, therefore the safety as well as the efficacy in this patient group is not clear.

Complement inhibition may be a useful adjuvant therapy in treating severe sepsis to reduce secondary damage from the inflammatory host response. However, clinical studies in (neonatal) bacterial sepsis are lacking, so the beneficial effect in (preterm) infants needs to be further explored.

## Neutrophil number and function

### Neutrophil number

Neutrophils are key players of the innate immune system, as they are important in opsonophagocytic killing of pathogens (68). Neutropenic patients are at risk for severe bacterial and fungal infections (69). Preterm neonates and especially very low birth weight neonates, have decreased neutrophil numbers, and thus, correcting neutropenia has long been a target for preventative and therapeutic strategies (70, 71). Several intervention studies, not including RCTs, have been performed. One strategy is stimulating the bone marrow to increase neutrophil production using granulocyte colony-stimulating factor (G-CSF). Although the use of G-CSF is widely used in neutropenic patients after chemotherapy, studies investigating the role of G-CSF in preventing neonatal sepsis show disappointing results thus far (72, 73). In most studies, administration did not lead to an increase in granulocytes and in the studies that did report an elevated absolute neutrophil count, there was no decrease in mortality (74, 75).

Instead of stimulating granulocyte production, another possibility is the direct transfusion of donor granulocytes (GTX). In clinical trials however, a preventative as well as a therapeutic strategy did not lead to a decrease in serious infections or mortality in patients with neutropenia or neutrophil dysfunction (76, 77). Another systematic review assessed the use of GTX for confirmed or suspected sepsis, specifically in neonates (78). There was no reduction in all-cause mortality when GTX was compared to placebo or no GTX. Compared to IVIG, GTX showed a reduction in all-cause mortality, which was borderline significant (p=0.06). So, although promising, transfusion of granulocytes did not lead to a significant decreased infection or mortality.

### Neutrophil function

Several studies document decreased function of neonatal neutrophils. Neutrophil extracellular trap (NET) formation is impaired in both preterm and term infants (79). In addition, neutrophil oxidative burst response is decreased in neutrophils of preterm infants when compared to term infants (80). Lastly, migration and chemotaxis of neutrophils is also impaired (81). Limited studies are available on improving neutrophil function of (preterm) neonates. One study showed that administration of G-CSF in neutropenic very low birth weight infants not only resulted in an increased absolute neutrophil count, but also improved oxidative burst (82). However, as discussed previously, administration of G-CSF does not improve mortality in neonatal sepsis.

When it comes to phagocytosis, no major impairment in bactericidal activity is evident in preterm and term neonates. Neutrophils of preterm infants are equally capable of bacterial phagocytosis of bacteria as term infants (6). This leads to the hypothesis that neonatal neutrophils could potentially be activated by external stimuli such as mAbs.

## Conclusion

Transient immunodeficiency of the newborn remains a significant risk factor for developing potentially life-threatening infections in preterm but also in term newborns. The development of protective and therapeutic treatment options is an ongoing challenge, but many promising strategies are currently being investigated. Presently, antibody supplementation therapy seems to be the closest to being a successful clinically applicable treatment, either in the form of maternal vaccination or mAbs (83). While maternal vaccination currently is the best strategy for term-born neonates, preterm neonates may benefit most from innovative antibody therapies, especially intravenous immunoglobulin G enriched with IgM and IgA, and modified synthetic polyvalent immunoglobulins (83).

Inhibition of complement activation in newborns may be beneficial to reduce organ dysfunction resulting from sepsis associated inflammation, but clinical studies are needed to see if this strategy benefits newborns with sepsis. Most of the strategies described in this review are focused on prevention of neonatal sepsis, but if successful, they could potentially also contribute to treatment.

Overall, neutrophils from term newborns seem sufficiently effective in opsonophagocytic killing, despite being reduced in number and decreased in effector functions. Opsonophagocytosic killing can be boosted either by immunoglobulins or potentially by complement activating treatment strategies.

The strength of this review is that it provides an overview of key aspects of neonatal and infant immunodeficiency and preclinical as well as clinical data on potential treatment interventions against infection. A limitation of this review is the focus on only three key elements of the neonatal immune system, not discussing the relevance of other deficiencies such as T-cell immunity. A second limitation is the scarcity of pre-clinical and clinical studies for most of the approaches discussed, making it difficult to predict efficacy of interventions in the context of the (preterm) neonatal immune system.

Future research should focus on clinical applicability of these promising prevention strategies for (preterm) neonatal sepsis.

## Author contributions

Conception and design: CB and MF. Preparation of the manuscript: CB. Literature review: CB. Preparation of figure: CB. Supervision: MF. Revision of the manuscript: DV, JM, SR, and MF. All authors contributed to the article and approved the submitted version.

## Funding

Wilhelmina Children’s Hospital Fund.

## Conflict of interest

The authors declare that the research was conducted in the absence of any commercial or financial relationships that could be construed as a potential conflict of interest.

## Publisher’s note

All claims expressed in this article are solely those of the authors and do not necessarily represent those of their affiliated organizations, or those of the publisher, the editors and the reviewers. Any product that may be evaluated in this article, or claim that may be made by its manufacturer, is not guaranteed or endorsed by the publisher.
