# Supplementary material for: Neonatal sepsis and transient immunodeficiency: Potential for novel immunoglobulin therapies?
Source: Front Immunol. 2022 Oct 18;13:1016877. doi: 10.3389/fimmu.2022.1016877 (PMC9623314; doi:10.3389/fimmu.2022.1016877)
Supplement: Supplementary file 1 [file DataSheet_1.pdf]

Supplementary Table 1. Key references to clinical studies aimed to improve outcome in (neonatal) infections

**Authors**	**Study design**	**Intervention**	**Target population**	**Main outcome(s)**
IVIG + IgM				
Ohlsonn et al (2020)	Cochrane systematic review	IgM enriched IVIG (Pentaglobin) vs placebo	Newborns with suspected sepsis	IgM enriched IVIG did not reduce mortality compared to placebo.
Welte et al (2018)	Randomized controlled trial	IgM enriched IVIG (Trimodulin) vs placebo	Adults with severe community-acquired pneumonia	IgM enriched IVIG did not reduce mortality compared to placebo. Post-hoc analysis did show improved outcome regarding mortality in patients with elevated CRP, reduced IgM or both in the Trimodulin group.
Maternal vaccination				
Simoes et al (2022)	Randomized controlled trial	RSV prefusion F (PreF) vaccine vs placebo	Pregnant women and their infants	Estimated vaccine efficacy of 84.7% in preventing any medically attended RSV-associated lower respiratory tract illness; 91.5% in preventing severe RSV-associated lower respiratory tract illness.
Madhi et al (2016)	Randomized controlled trial	Trivalent group B streptococcus (GBS) vaccine or placebo	Pregnant women and their infants	Maternal vaccination led to higher GBS serotype-specific antibody concentrations in infants than in placebo. Vaccine efficacy trials are ongoing.
Munoz et al (2014)	Randomized controlled trial	Tetanus Diphteria and Acellular Pertusis (TdaP) immunization vs placebo	Pregnant women and their infants	Maternal immunization with Tdap led to significantly higher concentrations of antibodies in infants until DTaP vaccination at age 2.
Madhi et al (2014)	Randomized controlled trial	Influenza vaccine vs placebo	Pregnant women and their infants	Influenza vaccine was immunogenic in HIV-uninfected pregnant women and provided partial protection against confirmed influenza in women and in infants.
Jackson et al (2011)	Randomized controlled trial	H1N1 influenza vaccine or hemagglutinin	Pregnant women and their infants	H1N1 influenza vaccine led to higher antibody titers in mothers as well as infants.
Monoclonal antibodies				
Griffin et al (2020)	Randomized controlled trial	Nirsevimab vs placebo	Late preterm infants	A single dose of nirsevimab led to fewer medically attended RSV-associated lower respiratory tract infections in healthy late preterm infants throughout the entire RSV season.

Weinreich et al (2019)	Randomized controlled trial	Casirivimab and imdevimab (REGEN-COV) vs placebo	Outpatient adults with Covid-19	Administration of a combination of casirivimab and imdevimab (REGEN-COV) in outpatients with Covid-19 led to fewer hospital admissions and reduced the viral load of SARS-CoV two more rapidly than placebo.
Wilcox et al (2017)	Randomized controlled trial	Bezlotoxumab vs actoxumab vs bezlotoxumab + actoxumab vs placebo	Adults with primary or recurrent *C. difficile*	Bezlotoxumab reduced the rate of recurrent infections of *C. difficile* compared to placebo. Actoxumab did not have an additional beneficial effect.
Patel et al (2015)	Expert opinion: randomized controlled trial	Prophylactic pagibaximab vs placebo	Very low birth weight preterm infants	There was no observed difference in sepsis cases or mortality in pagibaximab (anti-staphylococcal mAb) treated patients compared to placebo.
Anti-staphylococcal hyperimmune IgG				
Shah et al (2010)	Cochrane systematic review	Prophylactic INH-A2 vs placebo and Altastaph vs placebo	Very low birth weight preterm infants	There was no observed difference in sepsis cases or mortality in anti-staphylococcal HIG treated patients compared to placebo.
Complement inhibition				
Aurora et al (2022)	Case series	Eculizumab treatment	Pediatric patients with SARS-CoV-2 infection, multi-system inflammatory disease and thrombotic microangiopathy	Treatment with eculizumab led to improved outcome in three pediatric patients with SARS-CoV-2 infection, multi-system inflammatory disease and thrombotic microangiopathy.
Vlaar et al (2020)	Randomized controlled trial	Vilobelimab vs best supportive care	Adults with severe COVID-19	There was no difference in the main outcome of the study (change in PaO2/FiO2 after five days) between vilobelimab and best supportive care. A smaller proportion of patients in the vilobelimab group had pulmonary embolisms compared to the control group.
Increasing neutrophil numbers				
Carr et al (2003)	Cochrane systematic review	Prophylactic GM-CSF vs placebo	Newborns on intensive care	No significant reduction in mortality in newborns receiving GM-CSF

Estcourt et al (2015)-1	Cochrane systematic review	Prophylactic granulocyte transfusions (GTX) vs not receiving GTX.	Neutropenic patients	No significant reduction in all-cause mortality in patients receiving GTX vs not receiving GTX. There is low quality evidence of reduced incidence of bacteraemia and fungaemia in patients with myelosuppressive chemotherapy or stem-cell transplantation receiving GTX.
Estcourt et al (2015)-2	Cochrane systematic review	Therapeutic GTX vs no GTX	Neutropenic patients with infections	There is insufficient evidence to support a decrease in mortality in neutropenic patients with infections.
Pammi et al (2011)	Cochrane systematic review	GTX vs placebo<br>GTX vs IVIG	Infants with sepsis with and without neutropenia	No significant reduction in all-cause mortality in the GTX group vs placebo and a borderline significant reduction in all-cause mortality in the GTX group vs IVIG (P=0,06).

Supplementary Table 2. Key references to preclinical proof of concept studies aimed to improve host defense against (neonatal) infections

**Authors**	**Pathogen**	**Study type**	**Main outcome(s)**
Monoclonal antibodies			
Storek et al. (2018)	*E. coli*	In vitro serum bactericidal assay	A mAb targeted against the β-barrel assembly machine of E. Coli has a bactericidal effect.
Ali et al (2014)	*S. pneumoniae* and *N. meningitidis*	In vitro opsonization<br>In vitro serum bactericidal activity<br>In vivo murine sepsis model	Administration of properdin increased complement opsonization, serum bactericidal activity and bacterial clearance in a murine sepsis model.
Zwarthoff et al (2021)	*S. aureus*	In vitro complement activation and phagocytosis	Hexamer-enhancing mutations improve complement dependent phagocytosis of S aureus by human neutrophils compared to wild type antibodies.

Aguinagalde et al (2022)	*S. pneumoniae*	In vitro complement activation, phagocytosis and killing<br>In vivo murine model of passive immunization	Hexamerization-enhancing mutations improve complement activation and phagocytosis and killing by neutrophils in vitro and passive immunization protected mice from developing severe pneumonia.
Gulati et al (2019)	*N. gonorrheae*	In vitro serum bactericidal activity<br>In vivo murine gonorrhea model	Serum bactericidal activity of hexamerization enhancing Fc-mutant mAb 2C7 is significantly higher than WT Fc 2C7. Activation of the complement system is necessary for clearance of *N. gonorrheae,* neutrophils are not necessary. Administration of mAb 2C7 as a treatment led to bacterial clearance of *N. gonnorheae* in mice.
De Vor et al (2022)	*S. epidermidis*	In vitro phagocytosis and killing by healthy adult neutrophils + adult serum; phagocytosis by healthy adult neutrophils + neonatal plasma.	Hexamerization enhancing Fc-mutation E345K in several mAbs targeted against *S. epidermidis* led to more efficient phagocytosis of *S. epidermidis* by healthy adult neutrophils in the presence of adult serum as a complement source. The mAbs also significantly augmented phagocytosis of *S. epidermidis* by healthy adult neutrophils in the presence of neonatal plasma.
Complement enhancement			
Ali et al (2014)	*S. pneunoniae* and *S. meningitidis*	Therapeutic properdin administration in a murine sepsis model	Prophylactic properdin administration to mice infected with either *S. pneumonia* and *S. meningitidis* showed significant reduction of sepsis incidence compared to the control group.
